# Differential Matrix Rigidity Response in Breast Cancer Cell Lines Correlates with the Tissue Tropism

**DOI:** 10.1371/journal.pone.0006361

**Published:** 2009-07-23

**Authors:** Ana Kostic, Christopher D. Lynch, Michael P. Sheetz

**Affiliations:** Department of Biological Sciences, Columbia University, New York, New York, United States of America; Health Canada, Canada

## Abstract

Metastasis to a variety of distant organs, such as lung, brain, bone, and liver, is a leading cause of mortality in the breast cancer patients. The tissue tropism of breast cancer metastasis has been recognized and studied extensively, but the cellular processes underlying this phenomenon, remain elusive. Modern technologies have enabled the discovery of a number of the genetic factors determining tissue tropism of malignant cells. However, the effect of these genetic differences on the cell motility and invasiveness is poorly understood. Here, we report that cellular responses to the mechanical rigidity of the extracellular matrix correlate with the rigidity of the target tissue. We tested a series of single cell populations isolated from MDA-MB-231 breast cancer cell line in a variety of assays where the extracellular matrix rigidity was varied to mimic the environment that these cells might encounter *in vivo*. There was increased proliferation and migration through the matrices of rigidities corresponding to the native rigidities of the organs where metastasis was observed. We were able to abolish the differential matrix rigidity response by knocking down Fyn kinase, which was previously identified as a critical component of the FN rigidity response pathway in healthy cells. This result suggests possible molecular mechanisms of the rigidity response in the malignant cells, indicating potential candidates for therapeutic interventions.

## Introduction

Metastases cause about 90% of deaths from solid tumors and are very clinically diverse [1]. Metastasis occurs when cancer cells adapt to the microenvironment of an organ distant from the primary tumor and develop secondary tumors. Clinical observations indicate that certain organs are more susceptible to metastases. The specificity of the tumors for particular distant targets is known as tissue tropism. Although the first records of this phenomenon date from the late 19^th^ century, the mechanisms and molecular basis of tissue tropism are not fully understood. The visionary ‘soil and seed’ hypothesis of metastasis was introduced in 1889 by Stephen Paget who postulated that sites of secondary tumors are not randomly distributed throughout the body, and that some organs provide a more fertile environment than others for the growth of certain metastases [2]. Interestingly, this hypothesis has not been widely accepted, until its revival in 1980 by Ian Hart and Isaiah Fidler [3].

A number of recent studies have focused on characterization of genetic factors critical for tissue tropism of various tumors [4]–[9]. The most common target for breast cancer metastases is bone, followed by lung, liver, and brain [10], and distinct gene expression patterns have been associated with various metastases. Regardless of the tissue tropism, all metastatic tumors undergo the stage of dissemination to the regional lymph nodes. Through the lymphatic system, malignant cells reach distant organs where they can form secondary tumors. Biomechanical properties of the surrounding tissues, such as matrix composition and rigidity, are among the factors that govern this process. Despite numerous studies, their precise role remains poorly understood.

To identify genes critical in tissue tropism, the Massague laboratory used the MDA-MB231 breast cancer cell line, previously identified as highly invasive, to derive a number of single cell populations (SCPs) with different tumor target specificity [11]. The model used to determine tissue tropic orientation of individual SCPs involved injection of the cells in the cardiac ventricle, thereby circumventing the stage of the regional lymph node metastasis. The SCPs, isolated from the originally invasive line, displayed differential tissue tropism and affinity for specific secondary targets (lung, bone, adrenal glands) correlated with differential gene expression patterns [12], [13]. Interestingly, some of the SCPs have shown a clear preference for either bone, or lung, while others metastasized successfully to both target organs. Surprisingly, a number of SCPs displayed no or very little metastatic potential. Clusters of genes up/down-regulated in SCPs targeting a specific organ have been identified. Intriguingly, one of genes upregulated in bone-targeting SCPs was Fyn [12], a ubiquitously expressed Src-family kinase. We have previously identified this kinase as a critical signalling molecule in the fibronectin rigidity response in both fibroblasts and neurons [14], [15].

Here we report the differential rigidity response in various SCPs isolated from MBA-MD231 breast cancer cell line. The matrix rigidity selectively affected the cell proliferation and invasiveness in various SCPs. It appears that SCPs display increased proliferation and invasiveness in cases of matrix rigidity similar to the rigidity of *in vivo* target organs.

## Results

### Rigidity Dependence of proliferation for SCPs correlates with rigidity of the metastatic target

**Figure 1:**
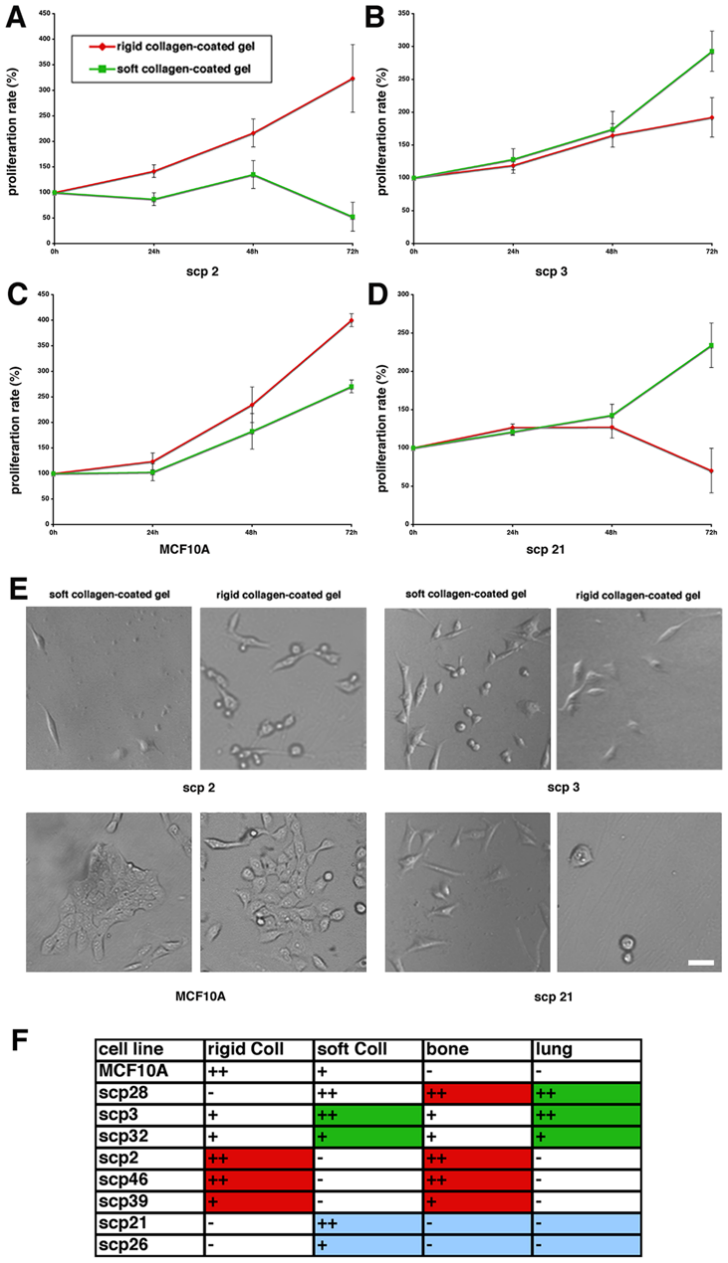
Collagen rigidity differentially affects proliferation of the various SCPs. (A-D) Breast cancer cell lines SCP 2, SCP3, SCP21, and breast epithelial cell lines MCF10A were plated on rigid and soft collagen-coated polyacrylamide gels and their proliferation was observed over the time period of 72 h. (E) Representative morphology of SCP 2, SCP3, SCP21, and MCF10A cells after 72 h incubation on soft and rigid collagen-coated gels. (F) Chart of the collagen rigidity response in SCP 2, SCP3, SCP21, SCP26, SCP28, SCP32, SCP39, SCP46, and breast epithelial cell line MCF10A and their specific *in vivo* tissue tropisms.

First, we tested whether proliferation of mammary control cells (MCF10A, hereafter), and various SCPs was affected by matrix rigidity. Cells were plated on collagen(Coll)-coated polyacrylamide gels, and the viable, adherent cells were counted each 24 h over the course of 72 h after plating (Fig. 1). As expected, the proliferation rate of control mammary cells was increased on rigid matrices compared to soft substrates. Further, SCPs displayed differential proliferation depending on rigidity of the matrix. The nonmetastatic SCPs, namely SCP21 and SCP26, proliferated only on soft Coll-coated gels, while rigid substrates did not support cell growth. The SCPs targeted specifically to the bone, such as SCP2, SCP46, and SCP39, showed preferential growth on rigid Coll-coated substrates, while soft substrates inhibited their proliferation. Interestingly, the SCP with high metastatic potential to both the bone and the lung, SCP28, proliferated only on soft Coll-coated gels. This result was difficult to interpret, and it could be explained by generally low proliferation rates displayed by SCP28 indicating distinct regulatory mechanisms of growth *in vitro* versus *in vivo* conditions. The SCPs that caused metastasis primarily in the lungs, but also, at lower frequency, to the bone, SCP3 and SCP32, proliferated faster on soft Coll-coated substrates than on rigid substrates. We conclude that collagen rigidity response in various SCPs correlates with the tissue tropism displayed *in vivo*.

### Effect of the rigidity of fibronectin-coated substrates on cell proliferation was similar to the effect of collagen rigidity

**Figure 2:**
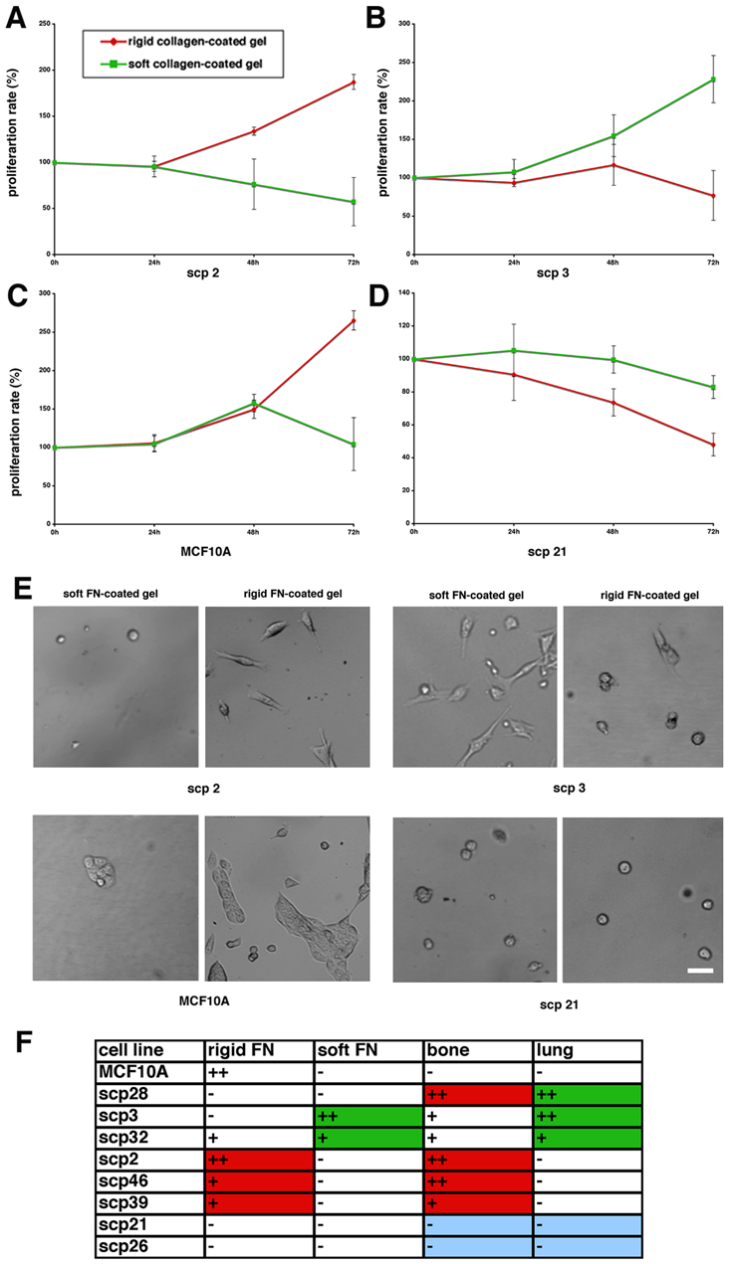
Fibronectin rigidity differentially affects proliferation of the various SCPs. (A-D) Breast cancer cell lines SCP 2, SCP3, SCP21, and breast epithelial cell lines MCF10A were plated on rigid and soft FN-coated polyacrylamide gels and their proliferation was observed over the time period of 72 h. (E) Representative morphology of SCP 2, SCP3, SCP21, and MCF10A cells after 72 h incubation on soft and rigid FN-coated gels (F) Chart of the FN rigidity response in SCP 2, SCP3, SCP21, SCP26, SCP28, SCP32, SCP39, SCP46, and breast epithelial cell line MCF10A and their specific *in vivo* tissue tropisms.

To determine the effect of the ECM composition on mammary cell proliferation, we tested proliferation of MCF10A cells and various SCPs on FN-coated polyacrylamide gels. The experiments were performed as in case of Coll-coated substrates, described above. Proliferation rate of control MCF10A cells was increased on rigid matrices, while being inhibited by soft FN-coated substrates (Fig. 2). Similar to Coll-coated substrates, SCPs proliferation rates were differentially affected by the rigidity of the FN substrate. However, some differences were observed. First, the nonmetastatic SCPs, SCP21 and SCP26, did not proliferate on FN-coated gels, regardless of their rigidity. Surprisingly, SCP28, characterized as highly metastatic to both target organs, displayed no growth on FN-coated substrates. Similar to the behavior observed on Coll-coated substrates, the SCPs targeted specifically to the bone, (SCP2, SCP46, and SCP39), displayed preferential growth on rigid FN-coated substrates, and proliferation was inhibited on the soft substrates. Further, the SCPs targeting primarily the lungs, and secondarily the bone, SCP3 and SCP32, grew better on soft FN-coated substrates than on rigid substrates.

### Fyn is required for rigid matrix-dependent proliferation of bone-targeted SCPs

**Figure 3:**
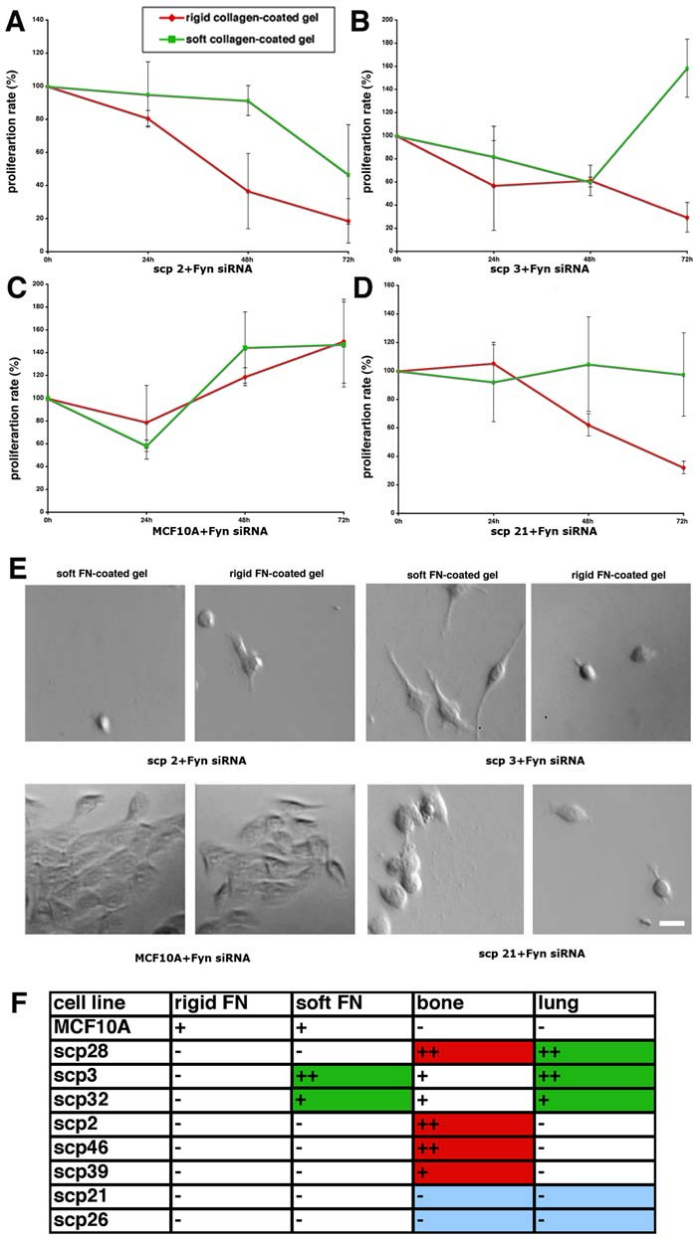
Fyn is required for survival of bone-specific SCPs on rigid FN-coated matrices. (A-D) Breast cancer cell lines SCP 2, SCP3, SCP21, and breast epithelial cell line MCF10A treated with Fyn siRNA were plated on rigid and soft FN-coated polyacrylamide gels and their proliferation was observed over the time period of 72 h. (E) Representative morphology of Fyn siRNA-treated SCP 2, SCP3, SCP21, and MCF10A cells after 72 h incubation on soft and rigid FN-coated gels (F) Chart of the effect of Fyn knockdown on the rigidity response in SCP 2, SCP3, SCP21, SCP26, SCP28, SCP32, SCP39, SCP46, and breast epithelial cell line MCF10A. Their original *in vivo* tissue tropisms are also shown.

To test the role of the Fyn in the matrix rigidity response of various SCPs, we used siRNA to knock down the expression of this kinase in all of the previously tested lines. While silencing of Fyn expression had no significant effect on proliferation of these cells on collagen-coated matrices (data not shown), the effect on the rigidity response on FN was striking (Fig. 3). Most importantly, Fyn siRNA-treated SCPs normally associated with bone metastasis, SCP2, SCP 46, and SCP39, lost their ability to proliferate on rigid FN-coated matrices, and their proliferation on soft matrices was also inhibited. The effect of Fyn siRNA on proliferation rate of other SCPs was less prominent, but an overall inhibitory effect was observed particularly on rigid surfaces. Importantly, lung-specific SCPs, SCP3 and SCP32, maintained high proliferation rates on the soft gels, despite the presence of Fyn siRNA. However, their normaly low rates of proliferation on rigid FN were further diminished. We speculate that the activity of Fyn kinase is required for activating cancer cell growth on rigid fibronectin matrices.

### The effect of 3-D matrix rigidity on cell invasiveness correlates with the tissue tropism

**Figure 4:**
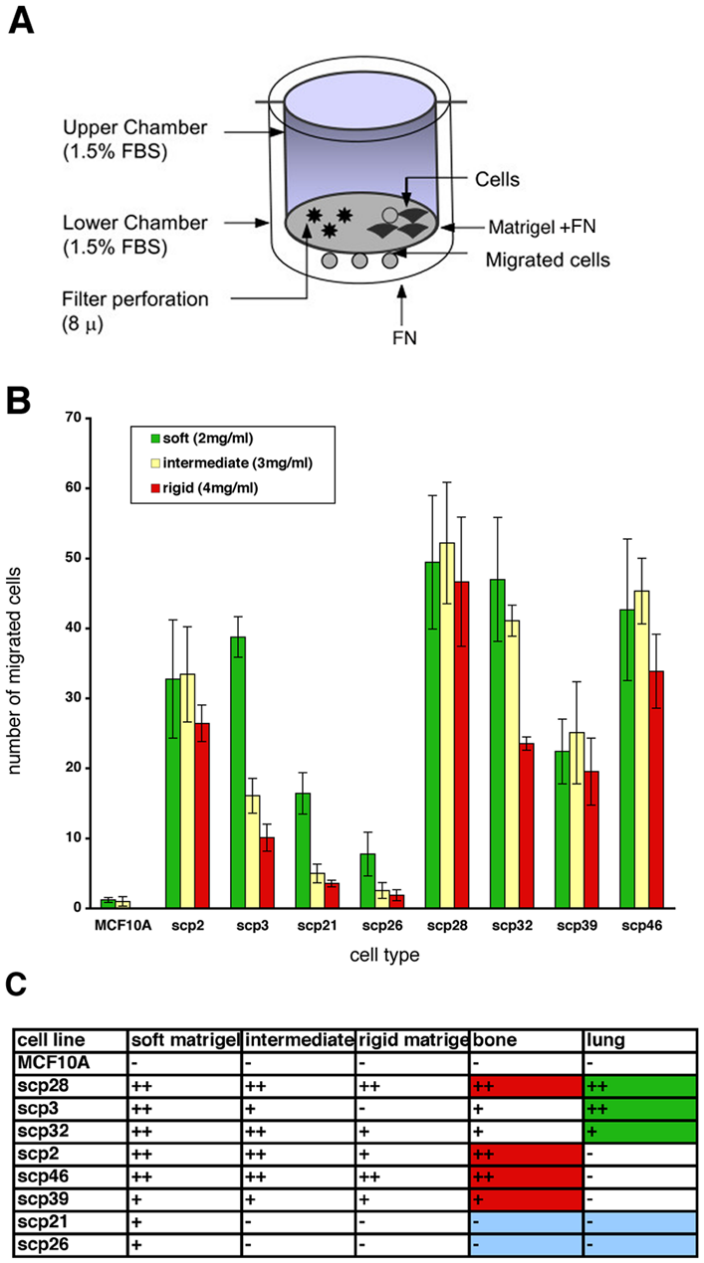
Transwell migration of various SCPs in differentially dependent on matrix rigidity. (A) Transwell system used consists of the upper chambers containg a thin layer of FN-enriched matrigel, perforated membrane, and the lower chamber coated with FN. (B-C) Breast cancer cell lines SCP 2, SCP3, SCP21, SCP26, SCP28, SCP32, SCP39, SCP46, and breast epithelial cell lines MCF10A were plated in matrigel pf increasing rigidities and their transwell migration rates were quantified after 48 h incubation.

The ability of the cancer cells to invade the surrounding tissues is critical in the process of metastasis. To test the invasiveness of the SCPs, we plated them on top of the 3-dimensional gels of varying rigidities. The gels were reconstituted from Matrigel and full-length fibronectin at the final concentration of 10 µg/ml (Materials and methods). The number of cells that invaded after 48 h was counted (Fig. 4) and compared for various SCPs. As expected, the control breast epithelial cells did not invade the gels regardless of the rigidity. Rigidity did not affect the invasiveness of the SCPs that preferably metastasized to the bone (SCP2, SCP46, SCP39, and SCP28). These cell lines displayed high levels of invasiveness regardless of the gel rigidity. The migration of SCPs that metastasized to the lung (SCP3, SCP32) was impeded by increasing gel rigidity. The cells lines that showed no metastatic potential (SCP26, SCP28), invaded poorly even through the gels of lowest rigidity. Therefore, we speculate that invasiveness of the SCPs is correlated with the tissue tropism *in vivo*.

### Early spreading events do not correlate with tissue tropism in SCPs

**Figure 5:**
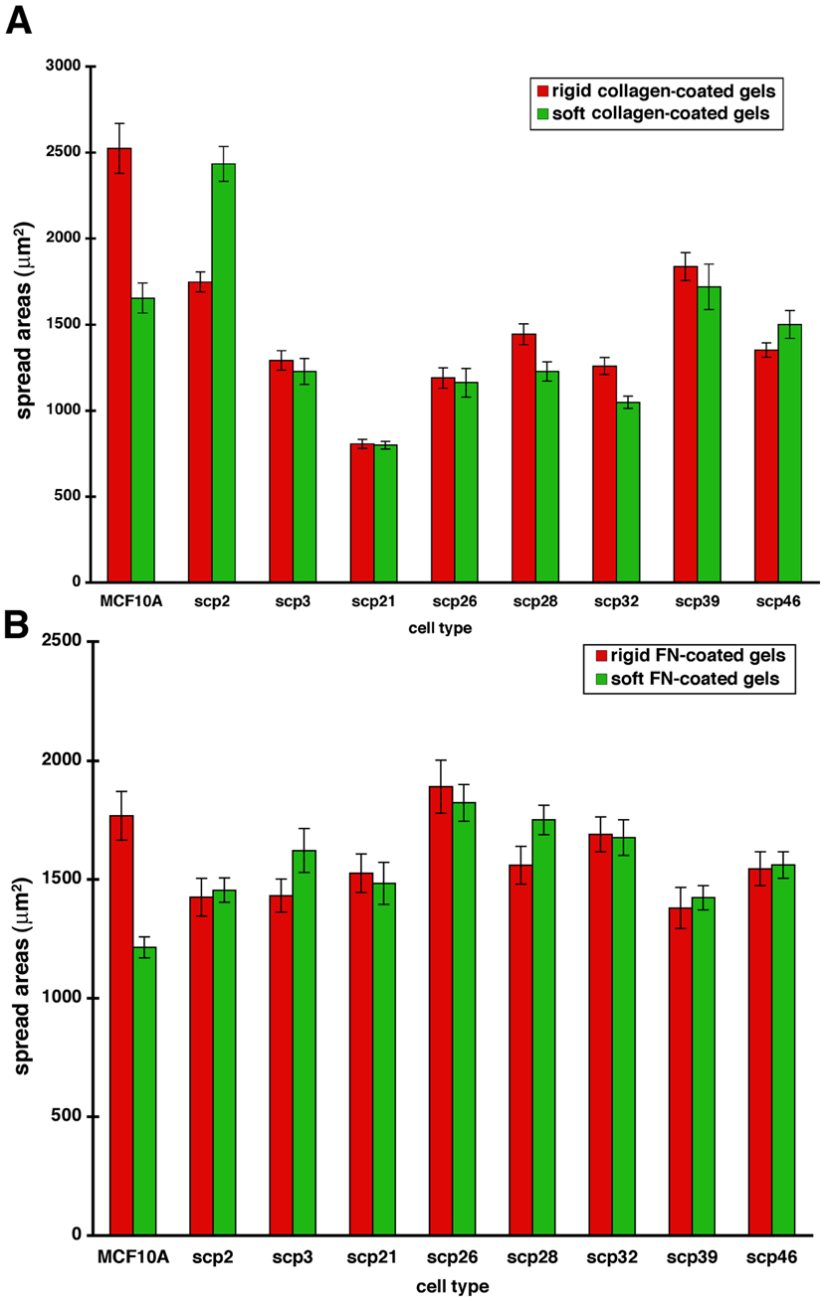
Matrix rigidity has no significant effect on spreading areas of the SCPs. (A-B) Breast cancer cell lines SCP 2, SCP3, SCP21, SCP26, SCP28, SCP32, SCP39, SCP46, and breast epithelial cell line MCF10A were allowed to spread on rigid and soft collagen-coated (A) or FN-coated (B) polyacrylamide gels for 2 h, when the spreading areas were quantified.

**Figure 6:**
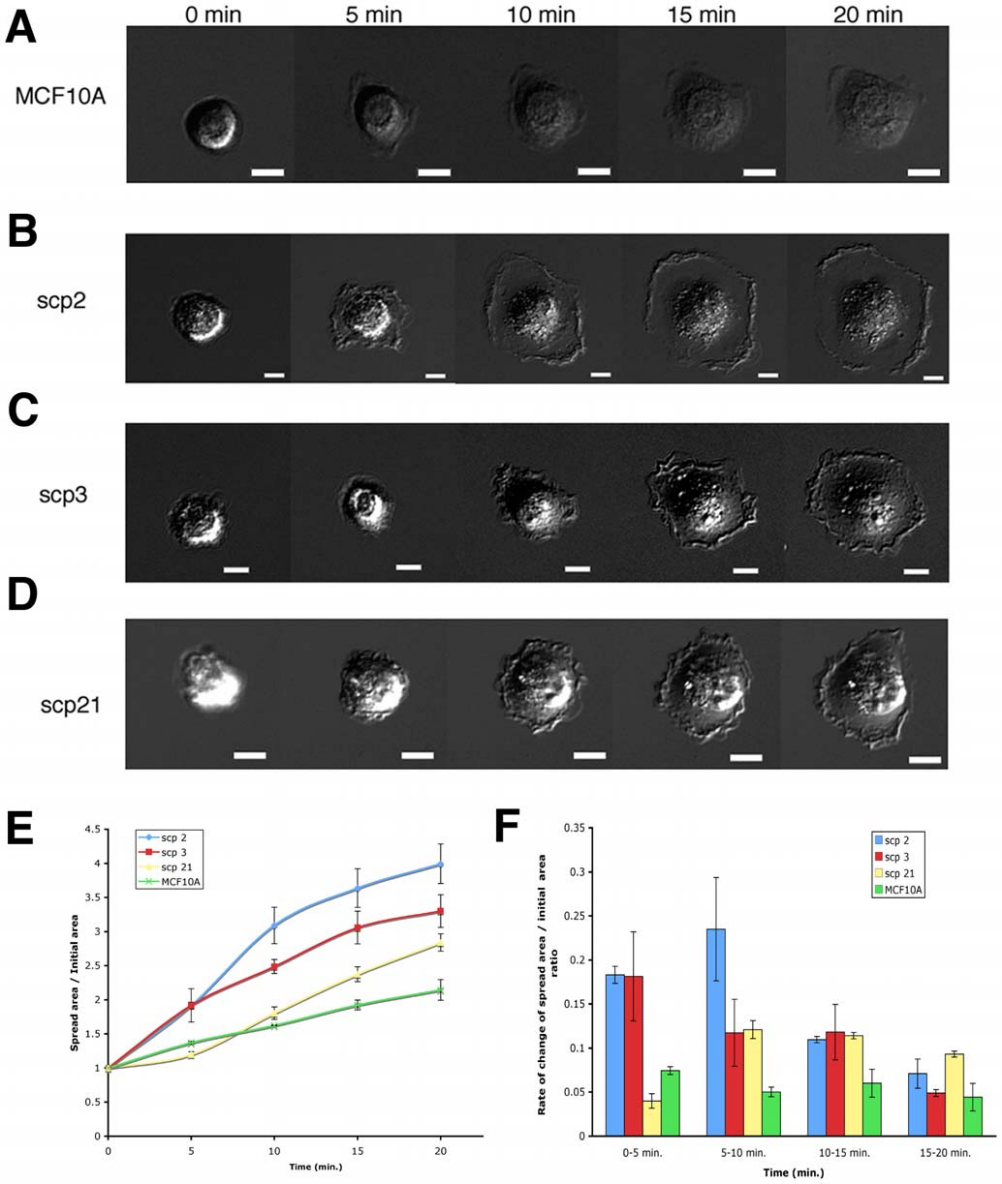
Early spreading motility appears uniform among different SCPs. (A-D) Breast epithelial cell line MCF10A (A), and breast cancer cell lines SCP 2 (B), SCP3 (C), and SCP21 (D) were plated on FN-coated glass and their spreading was recorded. The timecourse of representative cells is shown. No significant changes in cell area or motility were observed in cells that were observed longer than 20 min (up to 2 h). (E) Time dependence of the avareage spread area in SCP 2, SCP3, SCP21, and MCF10A cells. (F) Average spreading rates were calculated for 5 min time periods to quantify early spreading velocities in SCP 2, SCP3, SCP21, and MCF10A cells.

Early cell spreading on ECM-coated glass has been used as a model system for studying cell-ECM interactions [16], and it has been correlated to metastatic potential *in vivo* [17]. Therefore, we decided to quantify the effect of matrix rigidity on spreading of SCPs. Cells were incubated on FN/Coll-coated gels as described previously and their spread areas were quantified. Although we observed statistically significant differences from control cells, matrix rigidity did not appear to have an effect on spreading of various SCP lines (Fig. 5). However, it was possible that spread area reflected subtle differences in cell spreading processes that could account for differential ability to proliferate on rigid matrices displayed by bone-metastatic SCPs. Thus, we looked more closely at the spreading process on FN-coated glass. The time course of the early spreading events in representative cells (bone-specific SCP2, lung-specific SCP3, non-metastatic SCP21, and control MCF10A cells) was quantified (Figure 6). All SCPs exhibited high levels of ruffling around the edges of protruding lamellipodia indicating unstable focal complexes. However, all the cells spread relatively quickly to their final area (20–30 min), which was followed by periodic contractions. Interestingly, the metastatic lines displayed slightly increased spreading velocity compared to nonmetastatic and control lines. Cell spreading of SCPs was also tested on collagen-coated glass, and no significant differences were observed (data not shown). However, cell spreading on collagen was slower than spreading on FN (1–2 h), confirming the differential effect matrix had on cancer cell motility. Hence, we propose that cell spreading is not a particularly good indicator of the differential motility, proliferation, and invasiveness in the breast cancer cells.

## Discussion

Cellular microenvironment is critical in the orchestration of tumorogenesis and metastasis. Various factors present in the microenvironment, such as growth factor concentrations, homing receptors, matrix components, and mechanical properties of the matrix, have been shown to affect tumor growth and metastasis [18]. In this study, we focused on the effect of the mechanical rigidity of the ECM on proliferation and invasiveness of the breast cancer cells.

Mechanosensing is involved in cell motility, matrix remodeling, and development, as well as in a number of pathological processes, such as tumor formation and metastasis [19]. The rigidity of the extracellular matrix is one of the critical properties of the ECM and different cell types respond to matrix rigidity in fundamentally different ways [20]. The specific matrix rigidity requirements in different cell types have been correlated with the rigidities of their native tissues *in vivo* [21]. In addition, the differentiation of the stem cells through changes in gene expression was shown to depend on matrix rigidity [22]. Application of external mechanical force enhances integrin clustering and the subsequent recruitment of focal contact proteins, that, in turn, associate with the actin cytoskeleton and activate multiple signaling proteins including focal adhesion kinase (FAK), Src family kinases, Rho GTPase, and integrin-linked kinase (ILK), to promote cell growth, migration, and differentiation [23], [24]. In addition to the overall rigidity response being altered in malignantly transformed cells *in vitro* [25], similar behavior was described *in vivo* [26], [27] further emphasizing the relevance of proper mechanosignaling to complex processes such as differentiation, development, migration, and regeneration.

Interactions between epithelial cells and the extracellular matrix (ECM) regulate mammary gland development, and are critical for the maintenance of tissue homeostasis. The extracellular matrix regulates growth, survival, migration, and differentiation through a variety of transmembrane receptors. Similar to breast development, malignant transformation of the breast is also associated with significant alterations in ECM composition, turnover, processing, and orientation [24]. Intriguingly, certain epithelial cancers can be induced to regenerate normal tissue morphology when presented with embryonic mesenchyme or exogenous ECM scaffolds [18]. The upregulation of ECM genes has not only been detected in tumors, but has also been associated with poor prognosis [28]. In addition, cancer progression is characterized by extensive matrix remodeling and progressive stiffening of the stroma, which affects epithelial-stromal interactions, that could in-turn enhance epithelial cell growth, affect breast tissue organization, and promote cell invasion and survival. While the ECM in the normal breast is soft, the increased rigidity of the matrix, characteristic of malignant transformation, or externally applied force, stimulated proliferation and promoted a tumor-like behavior in mammary cells [25]. Furthermore, altered expression of β1-, β4-, α2-, α3- and α6-integrins has been reported in breast cancer cells [29].

In this study, we demonstrated a differential rigidity response in the single cell populations (SCPs) derived from a highly invasive breast cancer cell line MDA-MB231. These SCPs displayed differential metastatic potential and tissue tropism *in vivo*, which were correlated with different patterns of gene expression [12], [13]. Based on these profiles, SCPs displayed clustering in three groups that corresponded to their metastatic potential: bone, lung, or non-metastatic group. A few of these lines were also reported to cause adrenal gland metastases. Due to the intrinsic limitation of the animal model, no lines specifically targeting the brain or regional lymph nodes were isolated. As expected, different SCP clusters (bone-targeting, lung-targeting, and non-metastatic) showed differential rigidity responses on both collagen and fibronectin. While soft collagen matrices promoted proliferation of the lung-targeting and non-metastatic SCPs, rigid matrices inhibited growth of lung-targeting and non-metastatic lines, while promoting proliferation of bone-targeting SCPs. The effect of FN rigidity was even more pronounced, since most lines did not proliferate on FN-coated matrices, except bone-targeting lines that grew rapidly on rigid FN-coated gels. This observation can be correlated with the differences in the rigidity of the target tissues, which can have an effect on cell proliferation *in vivo*. Further, we showed that the ability of bone-targeting SCPs to proliferate on rigid matrices was critically dependent on activity of Fyn kinase, which has been previously detected as upregulated in these SCPs [12], [13]. This is interesting, especially in the light of our previous reports showing that Fyn kinase was indispensable in fibronectin rigidity sensing in fibroblasts and neurons [14], [15]. One can speculate that some of the rigidity pathways employed by healthy cells, can be misused in malignant cells. Next, we showed that the matrix rigidity affects the SCP's motility and invasiveness in the 3-D environment as well. Only bone-targeting lines were successful in migration through rigid matrigels, while lung-targeting SCPs invaded only through soft matrices, and non-metastatic lines showed no ability to invade. We speculate that matrix rigidity might similarly affect the cell invasiveness *in vivo*, which needs to be confirmed in the future studies. Finally, we are unable to detect any differences in early cell spreading and immediate rigidity response among various SCPs. This result can be interpreted by the differential effect that mechanical properties of the matrix have on cell spreading versus proliferation and invasiveness in transformed cells. Importantly, this study confirms the similarities between behavior of cancer cells in artificial matrix models and in the whole animal environment. Therefore, these systems could be used to further elucidate rigidity response mechanisms in cancer cells and potentially modulate these to develop novel diagnostic tools and therapeutic approaches.

## Materials and Methods

### Cell culture

MCF10A human breast epithelial cells and malignant SCPs isolated from MDA-MB-231 breast cancer line were generous gift from Professor Joan Massague. MCF10A cels were grown in 1∶1 mixture of DMEM and Ham's F-12 media supplemented with 5% Horse Serum, 100 µg/mL streptomycin, 2 mM L-glutamine, and 20 mM HEPES, 10 µg/ml insulin, 0.5 µg/ml hydrocortisone and 0.02 µg/ml EGF (all materials from Invitrogen). SCP cell lines were grown in high glucose DMEM supplemented with 10% Fetal Bovine Serum, 100 units/mL penicillin, 100 µg/mL streptomycin, 2 mM L-glutamine, and 20 mM HEPES.

### Spreading assays on polyacrylamide substrates

The full-length fibronectin (FN) or collagen (Coll)-coated polyacrylamide substrates were prepared as described previously [30]. The flexibility of the substrate was manipulated by maintaining the total acrylamide concentration at 5% while varying the bis-acrylamide component between 0.08% (rigid) and 0.03% (soft) (E = 3kPa, and E = 600Pa, respectively) [31]. The uniformity of ECM coating on the substrate surface was examined by coating the gels with FN/Coll conjugated to Cy5 fluorophore (Amersham Biosciences) according to manufacturer's instructions and visualized by confocal microscopy. Experiments were performed 2 h after the cells were plated on the polyacrylamide gels at a low density. Spread area was quantified for at least 50 cells for each condition and statistical significance of the results confirmed by t-test (p<0.01). Data is presented as mean±standard error.

### siRNA

*Silencer* ^TM^ siRNA transfection kit and custom siRNAs sequences targeted against Fyn (Ambion) were used according to manufacturer's protocol. The Fyn expression levels were tested by immunofluorescent staining with anti-Fyn (Chemicon) antibody. Controls with “scrambled” siRNA sequences were included.

### Antibodies

For this study, the following antibodies were used: a mouse monoclonal antibody against Fyn (Chemicon), an affinity purified polyclonal rabbit anti-phoshoY165Cas antibody (Cell Signaling Technology), HRP-conjugated anti-mouse and anti-rabbit antibodies (Amersham), an affinity purified rabbit polyclonal phosphoY416-Src kinase family antibody (Cell Signaling Technology), a goat anti-rabbit Ig conjugated with Alexa 647 (Molecular Probes), a goat anti-rabbit Ig conjugated with Alexa 555 (Molecular Probes), and goat anti-mouse Ig conjugated with Alexa 568 (Molecular Probes).

### Spreading assays on FN-coated glass

The glass coverslips were coated with 10 µg/ml full-length fibronectin (FN) for 1 h at 37°C. The cells were trypsynized, resuspended in serum-free DMEM media, incubated at 37°C, and then plated on FN-coated glass. Cell spreading was recorded for 30 minutes with a cooled CCD camera attached to an Olympus IX81 equipped with a 10× objective.

### Invasion assays

Gels were polymerized in the upper chamber of the transwells with polyester 8 µm-perforation membranes (Corning) (Fig. 3). By varying concentrations of Matrigel (BD Biosciences), that was reconstituted according to manufacturer's instructions at final concentrations of 2 mg/ml, 3 mg/ml, and 4 mg/ml, we obtained gels of varying rigidities. The full-length FN was added to the gels prior to the polymerization at the final concentration of 10 µg/ml. The bottom of the lower chamber was coated with 10 µg/ml FN to facilitate the adhesion of the invaded cells. Cells were plated on top of the gels, and cultured for 48 h, then fixed with 5% glutaraldehyde, and stained with toluidine blue to visualize the invaded cells. The number of invaded cells was counted using 20X objective. The minimum of 5 representative fields was counted for each condition [32].

### Immunocytochemistry

Mammary cells were plated onto FN/Coll-coated coverglasses (10 µg/mL FN/Coll) or FN/Coll-coated polyacrylamide gels. After incubation for the described time, cells were fixed in 3.7% formaldehyde and permeabilized with 0.1% Triton. Fixed cells were incubated with primary antibodies (described above) for 1 h followed by washing and incubation with appropriate fluorescent secondary antibodies (also described above). Fluorescent signals from all samples were visualized by confocal microscopy.

### Microscopy and analysis

Images of immunofluorescently stained samples were acquired using a Fluoview confocal microscope (Olympus, Melville, NY). Phase contrast images of the cells plated on polyacrylamide substrates were recorded with a cooled CCD camera attached to an Olympus IX81 equipped with a 10× objective. Analysis of acquired images was performed with the image analysis program, ImageJ (by W. Rasband (NIH, Bethesda, MD http://rsb.info.nih.gov/ImageJ).
